# Prenatal Diagnosis and Postnatal Outcomes of Fetal ADPKD: A Single-Center Retrospective Cohort Study

**DOI:** 10.3390/medicina61122145

**Published:** 2025-11-30

**Authors:** Suhra Kim, Ju-hee Yoon, Yun Ji Jung, Hayan Kwon, JoonHo Lee, Ja-Young Kwon, Young-Han Kim

**Affiliations:** Department of Obstetrics and Gynecology, Institute of Women’s Medical Life Science, Yonsei University College of Medicine, Yonsei University Health System, Seoul 03722, Republic of Korea; kimsuhra@yuhs.ac (S.K.); jhyoon95@yuhs.ac (J.-h.Y.); ccstty@yuhs.ac (Y.J.J.); whitekwon@yuhs.ac (H.K.); jleemd@yuhs.ac (J.L.); jaykwon@yuhs.ac (J.-Y.K.)

**Keywords:** autosomal dominant polycystic kidney disease, polycystic kidney disease, autosomal recessive polycystic kidney disease, chronic kidney disease, prenatal diagnosis, ultrasonography

## Abstract

*Background/Objectives:* Autosomal dominant polycystic kidney disease (ADPKD) is the most common hereditary renal disorder; it is typically diagnosed in adulthood, but occasionally presents antenatally as very-early onset ADPKD. Despite advances in prenatal ultrasonography, knowledge regarding the postnatal course of fetal ADPKD remains limited, restricting reliable prognostic assessment and prenatal counselling. This study aimed to evaluate the prenatal sonographic features of fetal ADPKD and their correlation with postnatal outcomes. *Materials and Methods:* We retrospectively reviewed 20 cases of prenatally suspected ADPKD diagnosed at a single tertiary referral center between 2006 and 2024. Prenatal ultrasonographic findings including renal size, cortical echogenicity, corticomedullary differentiation (CMD), and cortical cysts were analyzed and correlated with postnatal clinical and genetic outcomes. Postnatal follow-up data, including renal function and progression to chronic kidney disease (CKD), were collected with a median follow-up of 93.6 months. *Results:* The most consistent prenatal ultrasonographic findings were increased cortical echogenicity (85%), increased CMD (75%), and renal enlargement (35%), with cortical cysts detected in 45% of cases. Amniotic fluid volume was preserved in most cases (80%). Postnatally, most infants maintained normal or near-normal renal function, although two progressed to CKD. Both CKD cases demonstrated absent CMD on prenatal imaging. Sonographic features resembling autosomal recessive polycystic kidney disease (ARPKD) were associated with adverse outcomes. Although CMD severity showed no correlation with short-term neonatal outcomes, loss of CMD may still serve as a potential early indicator of long-term renal dysfunction. *Conclusions:* Fetal ADPKD was associated with heterogeneous postnatal outcomes. Loss of CMD and ARPKD-like sonographic appearances may be associated with adverse prognosis, whereas most infants maintained preserved renal function. Early recognition of ADPKD is crucial for accurate counselling, appropriate perinatal management, and long-term surveillance.

## 1. Introduction

Recent advances in prenatal ultrasonography, along with its widespread adoption in routine obstetric care, have increased the detection of fetal renal abnormalities. These abnormalities are now reported in approximately 10–20% of all prenatal ultrasound examinations [1].

Autosomal dominant polycystic kidney disease (ADPKD) is the most frequently encountered inherited renal disorder, affecting approximately 1 in 400–1000 individuals worldwide [2,3]. ADPKD contributes substantially to the global burden of kidney failure, accounting for up to 10% of end-stage kidney disease cases and serving as a major indication for kidney transplantation, which is performed in about two-thirds of affected patients [4].

ADPKD arises from pathogenic variants in two major genes: PKD1, located on chromosome 16p and responsible for the majority of cases (~85%), and PKD2, located on chromosome 4q and accounting for the remaining approximately 15% [5]. The PKD1 and PKD2 genes encode polycystin 1 and polycystin 2, which localize to the primary cilia of renal tubular epithelial cells and play essential roles in maintaining tubular architecture. variants in these genes impair ciliary function, resulting in dysregulated epithelial proliferation, increased fluid secretion, and cyst formation [6,7].

Although ADPKD is typically diagnosed in adulthood, it may occasionally present during childhood or even antenatally [8]. Such cases are classified as very-early onset ADPKD (VEO-ADPKD), defined by diagnosis before 18 months of age. VEO-ADPKD constitutes a rare and distinct clinical subset. This category further encompasses fetal ADPKD, which represents the earliest manifestation of the disease. Infants and young children with VEO-ADPKD are predisposed to early onset hypertension and show a greater likelihood of progression to chronic kidney disease (CKD) in childhood than do those diagnosed later [9,10,11,12,13,14].

The Kidney Disease: Improving Global Outcomes (KDIGO) 2025 Clinical Practice Guideline for ADPKD emphasized the importance of early recognition and longitudinal monitoring of atypical and pediatric forms of the disease [4]. The guideline newly introduced structured recommendations for antenatal and childhood diagnosis. It notes that the disease may begin earlier than traditionally recognized, including during the antenatal period, and that structural renal alterations are often detectable before any clinical signs become apparent. This recommendation underscores the clinical relevance of timely identification, as early detection enables individualized follow-up, anticipatory management of hypertension, and informed family counseling.

Despite nearly two decades of advances in prenatal imaging, information on the postnatal course of fetal ADPKD remains limited. Since a pivotal ultrasonographic study published in 2004, only a few subsequent reports have been published [13,15], restricting reliable prognostic assessment and prenatal counseling.

This study retrospectively reviewed a single-center cohort of patients with prenatally suspected ADPKD and evaluated their postnatal clinical and genetic outcomes. By correlating prenatal imaging findings with postnatal outcomes, the study aimed to improve diagnostic accuracy and provide evidence-based guidance for prenatal counseling.

## 2. Materials and Methods

### 2.1. Study Design and Procedure

This retrospective study was conducted at a tertiary referral center. Medical records of fetuses with renal abnormalities detected on prenatal ultrasonography and subsequently highly suspected or confirmed to have ADPKD were reviewed. ADPKD was diagnosed when prenatal ultrasonography findings alone were strongly indicative of the disease or when imaging findings raised a strong suspicion later confirmed by genetic testing or family history. Fetuses with no positive family history or genetic confirmation, but with the typical imaging features consistent with ADPKD, were classified as possible ADPKD (marked with an asterisk (*) in Table 1. These imaging criteria included the presence of bilateral multiple cortical cysts, commonly accompanied by bilateral renal enlargement and/or increased cortical echogenicity, without any features suggestive of other forms of cystic kidney disease [4]. Representative ultrasound images supporting diagnoses of possible ADPKD are presented in Supplementary Figures S1–S10. To ensure adequate image quality, only cases evaluated between 2006 and 2024 were included in the study. Clinical data, including prenatal sonographic findings, postnatal management, and outcomes of 20 fetuses with ADPKD, were extracted from electronic medical records and analyzed. This study was approved by the Institutional Review Board (YUHS 4-2025-0965) and conducted in accordance with the Declaration of Helsinki.

All prenatal ultrasonographic evaluations were performed by maternal–fetal medicine (MFM) specialists, MFM fellows, and expert sonographers with more than 10 years of experience in prenatal diagnosis. Scans were obtained using a multiplanar approach employing 2–7 MHz transabdominal transducers on Accuvix V20, WS80A, and HERA W10 (Samsung Medison, Seoul, Republic of Korea), Voluson 730 and Voluson E10 (GE Healthcare Ultrasound, Milwaukee, WI, USA), and iU22 (Philips Ultrasound, Bothell, WA, USA) ultrasound systems.

Prenatal ultrasonographic findings considered suggestive of ADPKD included the following: (1) normal or increased renal size, (2) increased cortical echogenicity, (3) increased corticomedullary differentiation (CMD), and (4) the presence of cortical cysts [15]. Prenatal data extracted from electronic medical records included maternal age, gestational age at diagnosis, ultrasound findings, and associated anomalies. The following parameters were recorded during ultrasound examinations: (1) renal length (centiles) [16], (2) cortical echogenicity (relative to the liver or spleen), (3) CMD (increased, decreased, or reversed), and (4) presence and location of renal cysts. Two obstetricians with specialised training in fetal ultrasound independently evaluated the presence of fetal ADPKD without access to the clinical information. In case of any disagreement, a third obstetrician with more than 20 years of experience was consulted for further evaluation.

Postnatal clinical, imaging and genetic data were also reviewed. Postnatal ultrasonographic findings of ADPKD were assessed based on the KDIGO diagnostic criteria [4].

**Figure 1:**
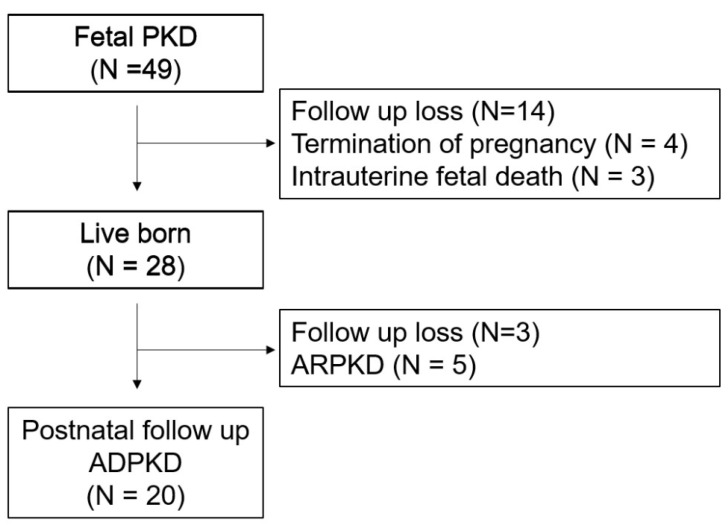
Study cohort selection. Forty-nine fetuses were prenatally suspected of having polycystic kidney disease (PKD). Fourteen were lost to prenatal follow-up, four pregnancies were electively terminated, and three resulted in intrauterine fetal demise. Of the 28 infants delivered at our institution, three were lost to postnatal follow-up and five were diagnosed with autosomal recessive polycystic kidney disease (ARPKD). A total of 20 cases were ultimately included in the analysis. ADPKD, Autosomal dominant polycystic kidney disease.

During the study period, 49 fetuses were suspected of having polycystic kidney disease on prenatal ultrasonographic findings. Among these, 14 were lost to prenatal follow-up, 4 pregnancies were electively terminated, and 3 resulted in intrauterine fetal demise. Of the remaining 28 infants delivered at our institution, 3 were lost to postnatal follow-up, and 5 were diagnosed with autosomal recessive polycystic kidney disease (ARPKD). Ultimately, 20 cases were eligible for analysis (Figure 1).

### 2.2. Statistical Analysis

Quantitative variables are expressed as medians with ranges, whereas categorical variables are expressed as frequencies and percentages. All statistical analyses were conducted using IBM SPSS, version 31.0 (IBM Corporation, Armonk, NY, USA). The association between CMD severity and neonatal outcome was evaluated using Spearman’s rank correlation, chosen due to the non-normal distribution of the data, treating CMD as an ordinal variable (1 = normal, 2 = increased, 3 = reversed, 4 = absent). The relationship between CMD pattern and CKD was analyzed using odds ratios, with the Haldane correction applied for zero cells. Statistical significance was set at *p* < 0.05.

## 3. Results

Overall, 20 cases of fetal ADPKD were identified. The demographic characteristics and prenatal ultrasonography findings are summarized in Table 2. One case involved a monochorionic diamniotic twin pregnancy (cases 14 and 15). Four karyotypic analyses were performed prenatally, all of which were normal.

**Figure 2:**
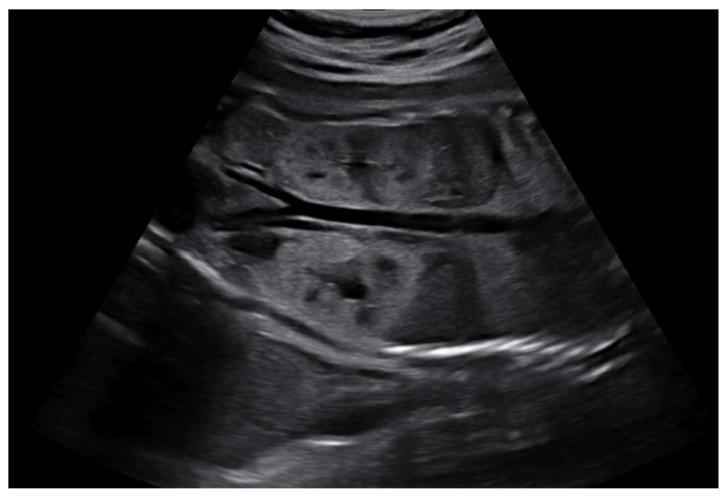
Sonographic findings of fetal ADPKD (Case 14). Ultrasound image of the first fetus of a monochorionic diamniotic twin pregnancy at 24 weeks’ gestation, demonstrating typical features of ADPKD. The kidney is enlarged above the 97th percentile, with a hyperechoic cortex and increased corticomedullary differentiation. Second fetus of the twin pregnancy showed same findings and diagnosis.

**Figure 3:**
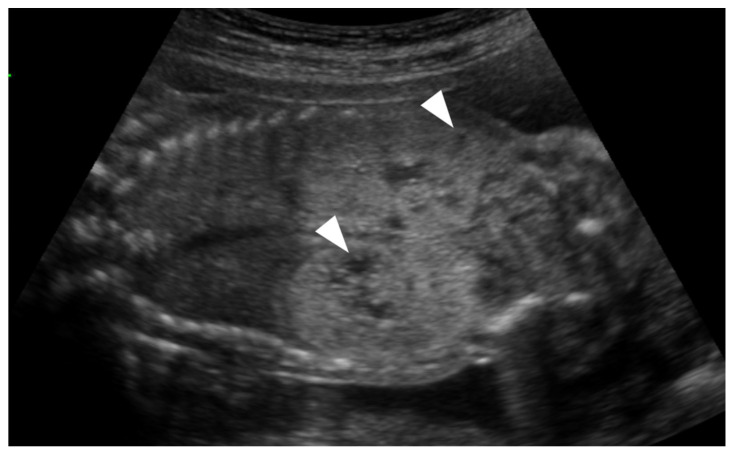
An unusual sonographic pattern of fetal ADPKD (Case 2). Ultrasound image at 22 weeks’ gestation showing markedly enlarged and diffusely hyperechoic kidneys with near-complete loss of corticomedullary differentiation. Small peripheral cysts are also visible (arrowhead). Although diagnosed as ADPKD, the imaging appearance closely resembles the sonographic pattern of ARPKD.

On prenatal ultrasonography, renal size above the 97th percentile was noted in 7 fetuses (35.0%). Increased cortical echogenicity compared with the liver or spleen was observed in 17 fetuses (85.0%). In 15 of these fetuses (75.0%), the medulla was hypoechogenic, resulting in increased CMD (Figure 2). In three fetuses (23%), the medulla was hyperechoic with decreased or absent CMD (Figure 3). Amniotic fluid volume was normal in 16 cases (80.0%), and the fetal bladder appeared normal in all cases. In one fetus (case 12) with anhydramnios, amnioinfusion was performed; reassessment at 36 weeks showed an amniotic fluid index of 14. Renal cysts were identified prenatally in 9 cases (45.0%).

Postnatal outcomes are summarized in Table 3. The median gestational age at delivery was 38 weeks and 0 days (range, 34 + 0 weeks to 41 + 1 weeks). Fetal growth restriction was not observed. Intubation was required in three cases, and five infants were delivered preterm. The mean postnatal follow-up period was 93.6 months (range, 15–216 months).

**Figure 4:**
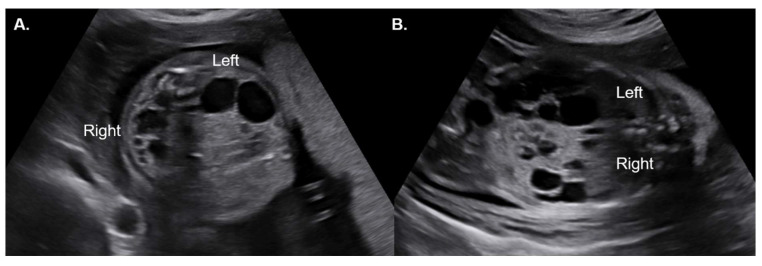
Imaging findings of a rare combined case of left MCDK and right ADPKD (Case 13). Ultrasound images demonstrating a multicystic dysplastic kidney (MCDK) on the left and ADPKD on the right. The ADPKD kidney shows multiple cysts of varying sizes, whereas the MCDK is characterized by larger, non-communicating cysts. (**A**) Axial plane; (**B**) Coronal plane.

Among the 20 cases of fetal ADPKD, we identified one rare case presenting with ADPKD in one kidney and multicystic dysplastic kidney (MCDK) in the contralateral kidney (Figure 4). CKD was developed in two cases (first and second cases). Both cases showed absent CMD on prenatal ultrasound. In case 1, the clinical course included dialysis, nephrectomy, and subsequent kidney transplantation. Because CKD occurred exclusively in the absent CMD group, the crude odds ratio was infinite, and the Haldane-corrected odds ratio was 185.0, suggesting a strong potential association between absent CMD and adverse renal outcome. However, given the small sample size and the absence of events in the comparison group, these findings should be interpreted with caution and considered exploratory.

Amniotic fluid volume was either reduced or within the normal range in both CKD cases. No significant correlation was observed between CMD severity (1 = normal, 2 = increased, 3 = reversed, 4 = absent) and NICU length of stay (Spearman’s *ρ* = −0.16, *p* = 0.49), and this result was unchanged after exclusion of an outlier. In addition, a weak positive correlation was found between CMD severity and 1-min Apgar score < 7 (*ρ* = 0.275, *p* = 0.240), although this association was not statistically significant.

In summary, these findings suggest that while CMD severity was not associated with short-term neonatal outcomes such as NICU stay and Apgar score, loss of CMD may serve as a potential indicator of long-term renal dysfunction.

Case-by-case details of the 20 fetuses are provided in Table 1. In addition, parental genetic testing identified a PKD1 variant in four families (cases 2, 12, 14, and 15). For comparison, five fetuses with ARPKD were also identified, and their characteristics are summarized in Supplementary Table S1.

## 4. Discussion

This retrospective study evaluated the prenatal and postnatal outcomes of fetuses diagnosed with ADPKD at a single tertiary referral center. The most characteristic prenatal sonographic findings included increased cortical echogenicity, altered CMD, and renal enlargement, with cortical cysts also identified in some fetuses. On postnatal follow-up, most infants maintained normal or near-normal renal function as assessed by creatinine clearance. However, early impairment, such as reduced glomerular filtration and CKD, was observed in a few infants. These findings suggest that prenatal diagnosis of ADPKD is associated with heterogeneous postnatal outcomes, highlighting the potential prognostic value of early imaging features.

Previous studies have emphasized the rarity of prenatal ADPKD, with most reported cases categorized as VEO-ADPKD. Earlier reports similarly described increased renal echogenicity and abnormal CMD as the most reliable prenatal sonographic features, which aligns with our study [15,17]. Brun et al. demonstrated that these features were commonly observed in fetuses subsequently confirmed to have ADPKD. Although cortical cysts are less commonly visualized in the prenatal period, they have been reported in selected cases, concordant with our findings [15].

Postnatal outcomes indicate that children diagnosed antenatally or in infancy are at an increased risk of early hypertension and progressive renal impairment [6]. Our data support this observation, as two infants in our cohort developed early renal dysfunction, whereas most maintained overall preserved function. Collectively, these findings indicate that absent CMD and sonographic features resembling ARPKD in ADPKD may serve as early indicators of an adverse prognosis. Because of the similarity in imaging findings, differentiating ADPKD from ARPKD remains critical. To illustrate this difficulty, we also included five ARPKD cases in Supplementary Table S1, as distinguishing the two entities prenatally continues to be challenging. Notably, ARPKD is more frequently associated with severe oligohydramnios or anhydramnios, which may aid in differential diagnosis.

In our cohort, both cases that progressed to CKD exhibited a loss of CMD on prenatal ultrasound. One fetus had markedly small kidneys, whereas the other showed pronounced enlargement with features mimicking ARPKD. Similar cases have also been reported by Garel et al., in which three of four fetuses had enlarged kidneys above the 97th percentile and absent CMD, although none were associated with oligohydramnios or anhydramnios [18]. Importantly, while their study could not evaluate long-term outcomes because of pregnancy termination or perinatal loss, our cohort extends these observations by correlating specific prenatal sonographic abnormalities with longitudinal postnatal renal outcomes.

Loss of CMD on prenatal ultrasound has long been regarded as a sign of impaired renal development and has consistently been associated with adverse postnatal outcomes [19,20]. Fetuses with absent CMD are at an increased risk of early renal dysfunction and progression to CKD. In line with our findings, previous reports have similarly shown that all live-born infants who developed CKD exhibited loss of CMD. This supports our observation that loss of CMD is closely associated with adverse postnatal renal outcomes in ADPKD [15,21,22]. Although the precise mechanisms remain unclear, CMD loss in renal insufficiency has been associated with conditions such as glomerulonephritis, acute tubular necrosis, end-stage renal disease, and obstructive uropathy [23,24,25,26]. Pathophysiologically, this likely reflects disruption of the cortical–medullary architecture and alterations in water distribution between the cortex and medulla, corresponding to advanced tubulointerstitial involvement and impaired urine concentrating ability. These processes may explain the poor renal prognosis observed in fetuses with absent CMD.

Among our study cohort, we identified a rare case presenting with ADPKD in one kidney and multicystic dysplastic kidney (MCDK) in the contralateral kidney (Figure 4). To our knowledge, no fetal or pediatric cases have been reported in which MCDK affects one kidney and ADPKD the contralateral kidney. Rare adult cases have been described, and in such situations reduced compensatory capacity may accelerate renal deterioration. This underscores the importance of long-term surveillance and multidisciplinary counseling [27,28].

This study has several strengths. First, it was conducted in a large tertiary referral center with high delivery volumes, enabling systematic case accumulation over an extended period and reflecting advances in ultrasonographic techniques. Notably, most previous studies investigating prenatal and postnatal outcomes of ADPKD were conducted more than two decades ago. Second, unlike previous studies with limited follow up periods of 12 [15] or 76 months [13], this study achieved a median follow-up of 93.6 months, allowing a more comprehensive evaluation of disease progression. Third, genetic testing, including next-generation sequencing, was available in a subset of cases, enabling genotype–phenotype correlation. Collectively, these features permitted an integrated analysis encompassing prenatal imaging, molecular data, and long-term outcomes, which is rarely achieved in ADPKD research.

Nevertheless, several limitations should be acknowledged. Firstly, the sample size was relatively small, reflecting the rarity of prenatally-diagnosed ADPKD, and the retrospective design may have introduced selection and information bias. In addition, genetic analysis was not feasible in all cases. According to the KDIGO 2025 guideline, individuals showing a clinical spectrum consistent with ADPKD, even without genetic confirmation, are still regarded as having these diseases. In clinical practice, the diagnosis of ADPKD is usually based on characteristic imaging features, clinical presentation, and/or family history, rather than on genetic testing, which is not mandatory for all patients. Accordingly, in cases lacking genetic results, the presence of typical findings such as bilateral renal cysts, renal enlargement, hepatic cysts, or reduced kidney function confirms the diagnosis of ADPKD [4].

Despite these constraints, our findings contribute important evidence by linking prenatal ultrasonographic features with longitudinal renal outcomes, thereby informing prenatal counselling and neonatal management. Early diagnosis of ADPKD has clear clinical implications. It enables prompt postnatal evaluation of renal function, preparation for neonatal intensive care if required, and timely family counselling. Prenatal identification also facilitates parental decision-making and anticipatory guidance regarding the risks of hypertension and renal impairment. This study highlights the value of ultrasound in prognostic stratification, while suggesting that extended follow-up may facilitate more comprehensive evaluation of postnatal outcomes. Future studies involving larger, multicenter cohorts and integrated genetic analyses will be essential to refine prognostic markers and to develop more multidisciplinary management strategies for families affected by ADPKD.

## 5. Conclusions

In this retrospective cohort of 20 infants with prenatally diagnosed ADPKD, increased cortical echogenicity, abnormal CMD, and renal enlargement were identified as the most consistent sonographic features. Loss of CMD and ARPKD-like renal appearances may be associated with adverse outcomes, whereas most infants maintained preserved renal function. Early recognition of ADPKD is clinically important for parental counselling, neonatal preparedness, and long-term surveillance. Further studies, especially those with larger sample sizes, are required to confirm these observations and to better define their prognostic implications.

## Figures and Tables

**Table 1:** Summary of the clinical data, prenatal ultrasonographic findings, genetic results, and postnatal outcomes of 20 cases of fetal ADPKD.

Case No.	GA at Dx (wks)	GA at Del (wks)	FHx	BW (kg)	Prenatal Renal Ultrasound					AF	Assoc. Anomalies	PN FU (mo)	PN Cyst	Postnatal Genetic Results	Postnatal Outcome
						Echogenicity									
					Size	Cort.	Med.	CMD	Cyst						
1	22 + 3	37 + 2	0	2.56	-	+	+	Abst	+	-	-	129	+	Pathogenic, Chromosome 17q12 deletion PKD1, c.6868G > T, p.Asp2290Tyr	CKD 4 (3 mo PN), Allograft, UTI, HD, PD, CRBSI, peritonitis
2	22 + 0	39 + 1	M	2.62	+	+	+	Abst	+	N	-	190	-	PKD1, c.12010C > T, p.Gln4004Ter	CKD 3 (9 yr PN), UTI, Deflux inj. for VUR, Blt UCN, CBD dilat., Rt orchiopexy + hernioplasty
3	36 + 0	38 + 3	M	4.28	+	+	-	+	Abst	N	-	216	+	N/A	
4 *	23 + 3	40 + 4	0	2.83	N	+	-	+	+	N	-	180	+	N/A	
5	19 + 2	38 + 0	P	3.05	+	N	N	N	+	N	Lt duplic.	85	-	PKD1 (c.11498G > C, p.Arg3833Pro, possibly damaging) Pathogenic PKHD1 (c.2507T > C, p.Val836Ala, probably damaging)	Right nephrectomy, APN, VUR (Blt G5), Cut. vesicostomy + revision, Nephrocalc., Met. acidosis, Hypona
6 *	32 + 0	34 + 0	0	2.16	N	+	-	+	Abst	N	-	147	+	N/A	
7 *	31 + 0	38 + 4	0	3.08	N	+	-	+	Abst	N	-	152	+	PKD1 not detected	Rt orchiopexy + hernioplasty
8 *	30 + 6	37 + 4	0	2.83	N	+	-	+	Abst	N	-	15	+	N/A	
9	29 + 6	39 + 3	0	3.21	+	N	N	N	+	-	-	126	+	PKD1, c.10678G > A, p.Gly3560Arg	
10	28 + 1	37 + 3	0	2.82	N	+	-	+	Abst	Abst	-	24	+	PKD1, c.7223G > A, p.Arg2408His PKD2, c.1354A > G, p.Ile452Val PKD2, c.1546G > T, p.Val516Leu	
11 *	21 + 3	37 + 3	0	2.98	N	+	-	+	+	N	-	55	+	PKD1 not detected PRKCSH detected	APN
12	23 + 5	37 + 4	M	2.96	N	+	-	+	Abst	N	-	55	+	PKD1, c.4955T > A, p.Leu1652Gln	
13 *	24 + 6	36 + 6	0	2.97	+	+	-	+	+	N	Lt. MCDK	55	+	NGS & trio test: no associated variant was detected	VACTERL assoc. (subglottic stenosis, imperf. anus, blt SVC)
14	20 + 0	36 + 0	P	2.50	+	+	-	+	Abst	N	-	42	+	PKD1, frameshift variant, pathogenic, paternal (NM_001009944.3(PKD1):c.2040dup (p.Ala681CysfsTer33)	Twin (MCDA)–first-born baby
15	20 + 0	36 + 0	P	2.78	N	+	-	+	Abst	N	-	42	+	PKD1, frameshift variant, pathogenic, paternal (NM_001009944.3(PKD1):c.2040dup (p.Ala681CysfsTer33)	Twin (MCDA)-second-born baby
16	24 + 0	39 + 5	M	3.12	N	+	-	+	Abst	N	-	26	+	N/A	Nephrocalc.
17 *	30 + 1	36 + 5	0	2.53	N	+	-	+	+	N	Cardiomeg.	23	+	N/A	Sensorineural hearing loss
18 *	24 + 0	41 + 1	0	3.72	N	-	+	Rev	Abst	N	Blt polydact.	56	+	N/A	Nephrocalc., blt polydact. (hands/feet), optic atrophy
19 *	26 + 5	39 + 1	0	3.17	N	+	-	+	Abst	N	-	127	+	N/A	
20 *	17 + 4	38 + 4	0	3.07	+	+	-	+	+	+	-	127	+	N/A	APN
Sum.	24 + 0	38 + 0	7/20 (35%)	2.96	7/20 (35%)	17/20 (85%)	3/20 (15%)	15/20 (75%)	9/20 (45%)	16/20 (80%)	4/20 (20%)	93.6	18/20(90%)	8/20 (40%)	CKD, 2/20 (10%); dialysis, 1/20 (5%); nephrectomy, 1/20 (5%); Allograft, 1/20 (5%); VUR, 2/20 (10%); UTI or APN, 5/20 (25%); nephrocalc., 3/20 (15%); orchiopexy/hernioplasty, 2/20 (10%)

+, increased; -, diminished; GA, gestational age; N, normal; N/A, not applicable; M, maternal; P, paternal; Abst, absent; AF, amniotic fluid; wks, weeks; CMD, corticomedullary differentiation; MCDK, multicystic dysplastic kidney; CKD, chronic kidney disease; HD, hemodialysis; PD, peritoneal dialysis; CRBSI, catheter-related bloodstream infection; VUR, vesicoureteral reflux; CBD, common bile duct; UTI, urinary tract infection; APN, acute pyelonephritis; VACTERL, vertebral defects, anal atresia, cardiac defects, tracheoesophageal fistula, renal anomalies, and limb abnormalities; MCDA, monochorionic diamniotic; NGS, next-generation sequencing; dx, diagnosis; del, delivery; FHx, family history; BW, birth weight; kg, kilograms; FU, follow-up; mo, months; Rev, reversed; PN, postnatal; yr, year; Blt, bilateral; inj, injection; G, grade; Cut, cutaneous; Nephrocalc, nephrocalcinosis; Met, metabolic; Hypona, hyponatremia; assoc, associated; imperf, imperforated; Cardiomeg, cardiomegaly; polydact, polydactyly; duplic, duplication; Cort, cortex; Med, medulla; Sum, summary. Summary line indicates the proportion of fetuses (*n* = 20) exhibiting each feature. Asterisk (*) indicates possible ADPKD, defined as diagnosis based on imaging findings only (without family history or genetic confirmation).

**Table 2:** Prenatal characteristics and ultrasonography findings of fetal ADPKD.

Variables	*n* = 20
Maternal age (years)	32.9 (26–44)
GA at prenatal diagnosis (weeks)	24 + 0 (17 + 4–36 + 0)
Ultrasonography findings	
Laterality	
Both	16 (80.0%)
AFI	
Oligohydramnios	2 (10.0%)
Anhydramnios	1 (5.0%)
Hydramnios	1 (5.0%)
Increased renal size (>97 percentile)	7 (35.0%)
Increased cortical echogenicity	17 (85.0%)
CMD	
Increased CMD	15 (75.0%)
Reverse CMD	1 (5.0%)
Loss of CMD	2 (10.0%)
Presence of cortical cysts	9 (45.0%)

Data are presented as average (range) or number (%). GA, Gestational age; CMD, Corticomedullary differentiation; AFI, Amniotic fluid index.

**Table 3:** Summary of postnatal outcomes of fetal ADPKD.

Variables	*n* = 20
Gestational age at delivery (weeks)	38 + 0 (34 + 0–41 + 1)
Preterm delivery (<37 weeks)	5 (25.0%)
Birth weight (kg)	2.96 (2.16–4.28)
Birth weight < 2500 g	1 (5.0%)
1-min AS < 7	11 (55.0%)
5-min AS < 7	2 (10.0%)
NICU admission	17 (85.0%)
NICU length of stay (days)	17.7 (3–211)
Ventilatory support (intubation)	3 (15.0%)
Renal cysts	18 (90.0%)
Renal enlargement	7 (35.0%)
Postnatal follow-up period (months)	93.6 (15–216)
CKD	2
Dialysis	1
Nephrectomy	1
Kidney transplantation	1
Vesicourethral reflux	2
Urinary tract infection or Acute pyelonephritis	5
Nephrocalcinosis	3

Data are presented as average (range) or number (%). AS, Apgar score; NICU, Neonatal Intensive Care Unit; CKD, Chronic kidney disease.

## Data Availability

The datasets analyzed during the current study are available from the corresponding authors.

## Supplementary Materials

The following supporting information can be downloaded at: https://www.mdpi.com/article/10.3390/medicina61122145/s1, Figure S1: Case 4 (possible ADPKD); Figure S2: Case 6 (possible ADPKD); Figure S3: Case 7 (possible ADPKD); Figure S4: Case 8 (possible ADPKD); Figure S5: Case 11 (possible ADPKD); Figure S6: Case 13 (possible ADPKD); Figure S7: Case 17 (possible ADPKD); Figure S8: Case 18 (possible ADPKD); Figure S9: Case 19 (possible ADPKD); Figure S10: Case 20 (possible ADPKD); Table S1: Summary of clinical data, prenatal ultrasonographic findings, and genetic results of five cases with fetal ARPKD.

## Abbreviations

The following abbreviations are used in this manuscript:

ADPKD	Autosomal dominant polycystic kidney disease
ARPKD	Autosomal recessive polycystic kidney disease
VEO-ADPKD	Very-early onset autosomal dominant polycystic kidney disease
CKD	Chronic kidney disease
CMD	Corticomedullary differentiation
MFM	Maternal-fetal medicine
AFI	Amniotic fluid index
IUFD	Intrauterine fetal demise

## References

[1] Irfan A., O’Hare E., Jelin E. (2021). Fetal interventions for congenital renal anomalies. Transl. Pediatr..

[2] Ebrahimi N., Garimella P.S., Chebib F.T., Sparks M.A., Lerma E.V., Golsorkhi M., Ghozloujeh Z.G., Abdipour A., Norouzi S. (2024). Mental Health and Autosomal Dominant Polycystic Kidney Disease: A Narrative Review. Kidney360.

[3] Grantham J.J., Mulamalla S., Swenson-Fields K.I. (2011). Why Kidneys Fail in Autosomal Dominant Polycystic Kidney Disease. Nat. Rev. Nephrol..

[4] Kidney Disease: Improving Global Outcomes (KDIGO) ADPKD Work Group (2025). KDIGO 2025 clinical practice guideline for the evaluation, management, and treatment of autosomal dominant polycystic kidney disease (ADPKD). Kidney Int..

[5] Ong A.C., Harris P.C. (2015). A polycystin-centric view of cyst formation and disease: The polycystins revisited. Kidney Int..

[6] Al-Orjani Q., Alshriem L.A., Gallagher G., Buqaileh R., Azizi N., AbouAlaiwi W. (2025). Mechanistic insights into the pathogenesis of polycystic kidney disease. Cells.

[7] Reeders S.T., Breuning M.H., Davies K.E., Nicholls R.D., Jarman A.P., Higgs D.R., Pearson P.L., Weatherall D.J. (1985). A highly polymorphic DNA marker linked to adult polycystic kidney disease on chromosome 16. Nature.

[8] Nowak M., Huras H., Wiecheć M., Jach R., Radoń-Pokracka M., Górecka J. (2016). Autosomal Dominant Polycystic Kidney Disease Diagnosed In Utero: A Review. Ginekol. Pol..

[9] Shamshirsaz A.A., Reza Bekheirnia M., Kamgar M., Johnson A.M., McFann K., Cadnapaphornchai M., Haghighi N.N., Schrier R.W. (2005). Autosomal-dominant polycystic kidney disease in infancy and childhood: Progression and outcome. Kidney Int..

[10] Nowak K.L., Cadnapaphornchai M.A., Chonchol M.B., Schrier R.W., Gitomer B. (2016). Long-term outcomes in patients with very-early onset autosomal dominant polycystic kidney disease. Am. J. Nephrol..

[11] Fick G.M., Johnson A.M., Strain J.D., Kimberling W.J., Kumar S., Manco-Johnson M.L., Duley I.T., Gabow P.A. (1993). Characteristics of very early onset autosomal dominant polycystic kidney disease. J. Am. Soc. Nephrol..

[12] Sedman A., Bell P., Manco-Johnson M., Schrier R., Warady B.A., Heard E.O., Butler-Simon N., Gabow P. (1987). Autosomal dominant polycystic kidney disease in childhood: A longitudinal study. Kidney Int..

[13] Boyer O., Gagnadoux M.F., Guest G., Biebuyck N., Charbit M., Salomon R., Niaudet P. (2007). Prognosis of autosomal dominant polycystic kidney disease diagnosed in utero or at birth. Pediatr. Nephrol..

[14] Tee J.B., Acott P.D., McLellan D.H., Crocker J.F. (2004). Phenotypic heterogeneity in pediatric autosomal dominant polycystic kidney disease at first presentation: A single-center, 20-year review. Am. J. Kidney Dis..

[15] Brun M., Maugey-Laulom B., Eurin D., Didier F., Avni E.F. (2004). Prenatal sonographic patterns in autosomal dominant polycystic kidney disease: A multicenter study. Ultrasound Obstet. Gynecol..

[16] Chitty L.S., Altman D.G. (2003). Charts of fetal size: Kidney and renal pelvis measurements. Prenat. Diagn..

[17] Gyokova E., Hristova-Atanasova E., Odumosu E., Andreeva A. (2025). Prenatal diagnosis of autosomal dominant polycystic kidney disease: Case report. Reports.

[18] Garel J., Lefebvre M., Cassart M., Della Valle V., Guilbaud L., Jouannic J.M., Ducou le Pointe H., Blondiaux E., Garel C. (2019). Prenatal ultrasonography of autosomal dominant polycystic kidney disease mimicking recessive type: Case series. Pediatr. Radiol..

[19] Gupta A., Aneja A., Bahl N., Arora R., Nadir L., Saini P. (2024). Corticomedullary differentiation in fetal kidneys: A necessary evil?. J. Fetal Med..

[20] Duel B.P., Mogbo K., Barthold J.S., Gonzalez R. (1998). Prognostic value of initial renal ultrasound in patients with posterior urethral valves. J. Urol..

[21] Buffin-Meyer B., Klein J., Aziza J., Fernandez M., Feuillet G., Seye M., Buléon M., Fédou C., Camus M., Burlet-Schiltz O. (2025). Improved prenatal assessment of kidney disease using multiple ultrasound features. Nephrol. Dial. Transplant..

[22] Dienye B.E., Ugboma E.W., Emem-Chioma P. (2025). Ultrasound correlation of renal indices with creatinine levels in chronic kidney disease in a tertiary hospital, South Nigeria: A pilot study. J. Biomed. Adv. Clin. Res..

[23] Semelka R.C., Corrigan K., Ascher S.M., Brown J.J., Colindres R.E. (1994). Renal corticomedullary differentiation: Observation in patients with differing serum creatinine levels. Radiology.

[24] Lee V.S., Kaur M., Bokacheva L., Chen Q., Rusinek H., Thakur R., Moses D., Nazzaro C., Kramer E.L. (2007). What causes diminished corticomedullary differentiation in renal insufficiency?. J. Magn. Reson. Imaging.

[25] Kettritz U., Semelka R.C., Brown E.D., Sharp T.J., Lawing W.L., Colindres R.E. (1996). MR findings in diffuse renal parenchymal disease. J. Magn. Reson. Imaging.

[26] Chung J.J., Semelka R.C., Martin D.R. (2001). Acute renal failure: Common occurrence of preservation of corticomedullary differentiation on MR images. Magn. Reson. Imaging.

[27] Xu J., Chen D.P., Mao Z.G., Huang H.F., Xu C.M., Wang C.R., Jia W.P., Mei C.L. (2013). Autosomal dominant polycystic kidney disease with ectopic unilateral multicystic dysplastic kidney. BMC Nephrol..

[28] Amoah Y., Kyei M.Y., Mensah J.E., Palm B., Adrah H.K., Asiedu I. (2024). Autosomal dominant polycystic kidney disease with ectopic unilateral multicystic kidney: A case report. J. Med. Case Rep..

