# Supplementary material for: Prenatal Diagnosis and Postnatal Outcomes of Fetal ADPKD: A Single-Center Retrospective Cohort Study
Source: Medicina (Kaunas). 2025 Nov 30;61(12):2145. doi: 10.3390/medicina61122145 (PMC12734453; doi:10.3390/medicina61122145)
Supplement: Supplementary file 1 [file medicina-61-02145-s001.zip › medicina-4014326-supplementary.pdf]

SUPPLEMENTARY MATERIAL

Supplementary Table S1. Summary of clinical data, prenatal ultrasonographic findings, and genetic results of five cases with fetal ARPKD.

Case No.	GA at dx (wks)	GA at del (wks)	A/S (1, 5 min)	FHx	BW (kg)	Prenatal renal ultrasound						AF	PN FU (mo)	Genetic results	Postnatal outcome
								Echogenicity							
						Size	Lat.	Cort.	Med.	CMD	Cyst				
1	20+5	35+6	1,2	0	2.32	N	Both	-	+	reverse	-	Abst	14	PKHD1, c.1675C>T, p.Arg559Trp<br>PKD1, c.1601C>T, p.Pro534Leu<br>ASCL1, c.526G>C, p.Glu176Gln<br>LRP5, c.4619C>T, p.Thr1540Met<br>RET, c.341G>A, p.Arg114His	Death following 14 months in NICU care. CKD stage 4, Peritoneal dialysis, Hepatosplenomegaly, Lung fibrosis, Pulmonary hypoplasia, Pneumothorax, Pulmonary arterial hypertension, Respiratory failure, Fungal sepsis, Liver failure
2	26+4	35+4	4,6	M	2.26	+	Both	+	+	Abst	-	Abst	1 day	Pathogenic, PKHD1, c.1690C>T, p.Arg564Ter<br>LP, PKHD1, c.5455T>A, p.Cys1819Ser	Death following 1day in NICU care, Acute respiratory failure, Pulmonary hypoplasia
3	32+6	35+4	6,7	0	3.27	+	Both	+	-	+	+	6	175	N/A	CKD stage 4, Hypertension, Sepsis, Pneumonia, VUR, s/p Lt, Percutaneous nephrostomy, Metabolic acidosis, UTI, CBD dilatation, Laparoscopic splenectomy (infarction of spleen)
4	21+1	36+4	5,6	0	3.25	+	Both	+	-	+	+	3	24	PKD1, c.1233G>A, p.Glu411	CKD stage 3, Hypertension, APN, Pulmonary hypoplasia, Pneumothorax
5	26+5	36+1	3,6	M	2.89	+	Both	+	+	Abst	-	Abst	49 days	LP, PKHD1, c.11212_11213delAT, p.Ile3738SerfsTer19	Death following 49 days in NICU care, CKD stage 4, Hemodialysis, Hypertension, Pulmonary hypoplasia, Pneumothorax, Bilateral nephrectomy at 3 weeks after birth (bowel compression)

+, increased; –, diminished; GA, gestational age; N, normal; N/A, not applicable; A/S, Apgar score; M, maternal; Abst, absent; AF, amniotic fluid; CMD, corticomedullary differentiation; CKD, chronic kidney disease; VUR, vesicoureteral reflux; CBD, common bile duct; UTI, urinary tract infection; APN, acute pyelonephritis; LP, likely pathogenic; Lat., laterality; mo, months; wks, weeks; dx, diagnosis; del, delivery; FHx, family history; BW, birth weight; kg, kilograms; FU, follow-up; PN, postnatal; yr, year.

Supplementary Figure S1. Case 4 (possible ADPKD)

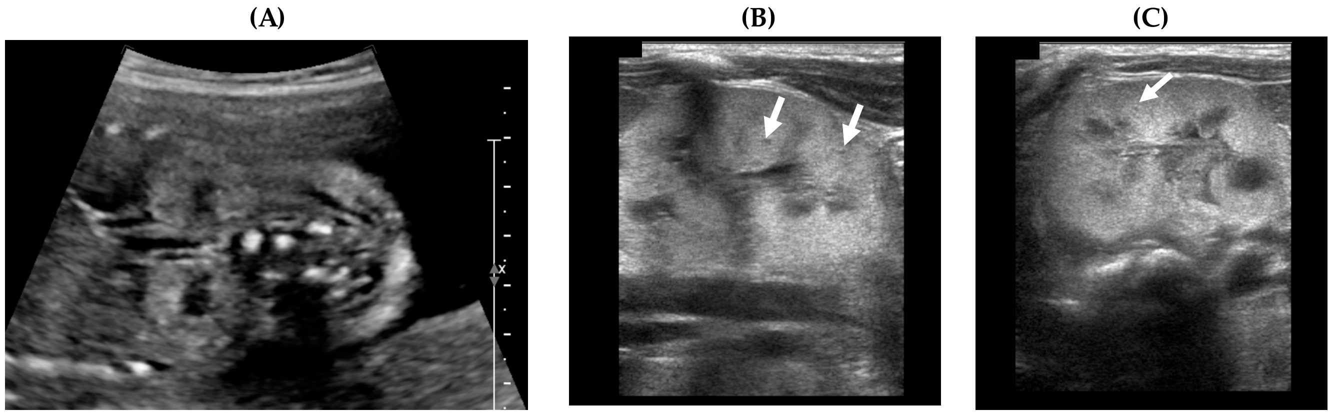

(A) Prenatal ultrasound shows bilaterally hyperechogenic kidneys.

(B, C) Postnatal renal ultrasound on the first day after birth demonstrates multiple bilateral cortical cysts and diffusely increased cortical echogenicity in both kidneys. (B) shows the right kidney, and (C) shows the left kidney. Arrows indicate renal microcysts.

Supplementary Figure S2. Case 6 (possible ADPKD)

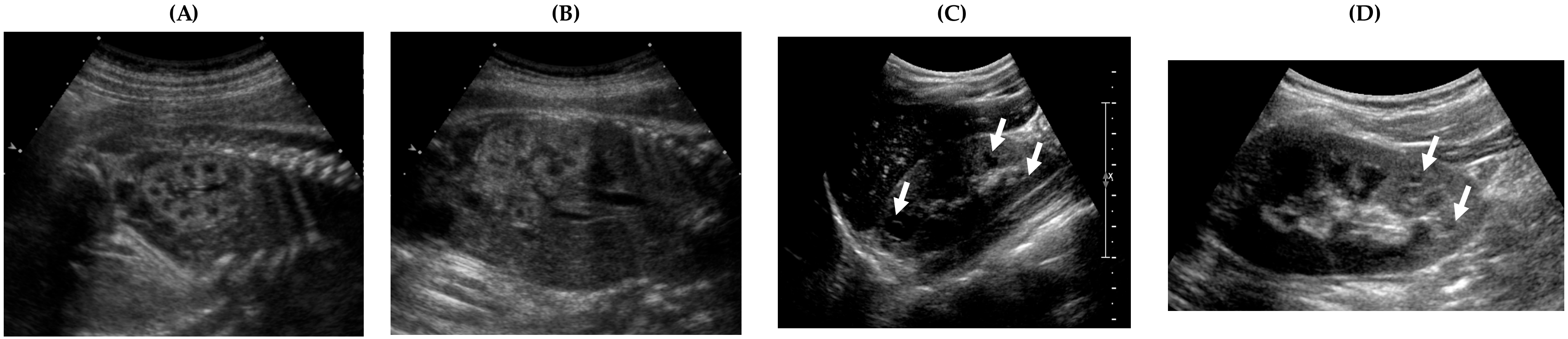

(A, B) Prenatal ultrasound indicates bilaterally hyperechogenic kidneys. (A) shows the right kidney, and (C) shows the left kidney.

(C, D) Postnatal renal ultrasound demonstrates diffusely increased cortical echogenicity and multiple cortical cysts in both kidneys, suggesting ADPKD. Arrows denote renal cysts.

Supplementary Figure S3. Case 7 (possible ADPKD)

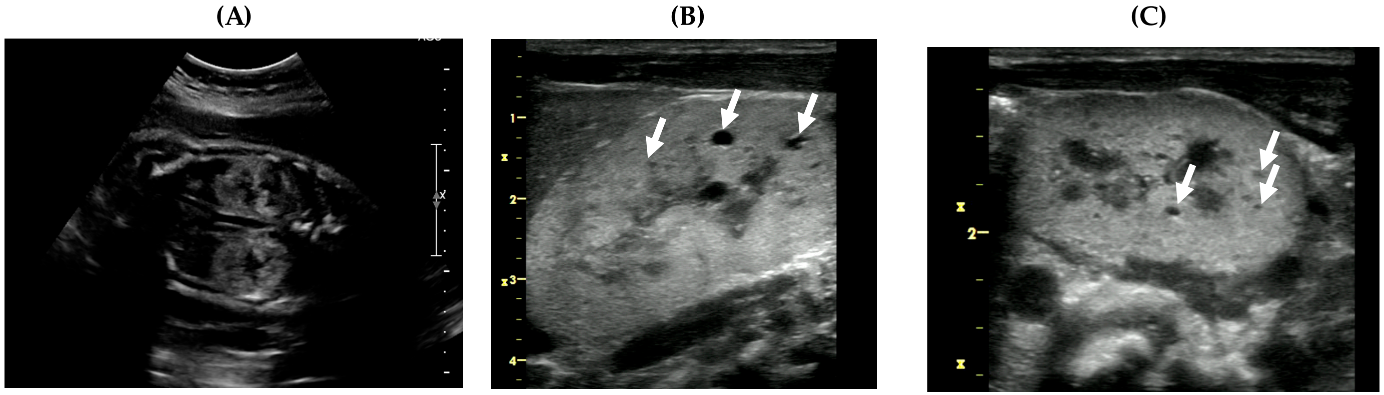

(A) Prenatal ultrasound shows bilaterally hyperechogenic kidneys.

(B, C) Postnatal renal ultrasound obtained on the day of birth demonstrates increased cortical echogenicity of both kidneys with multiple tiny cystic lesions in the renal cortex. (B) right kidney; (C) left kidney. Arrows mark cortical cystic lesions.

Supplementary Figure S4. Case 8 (possible ADPKD)

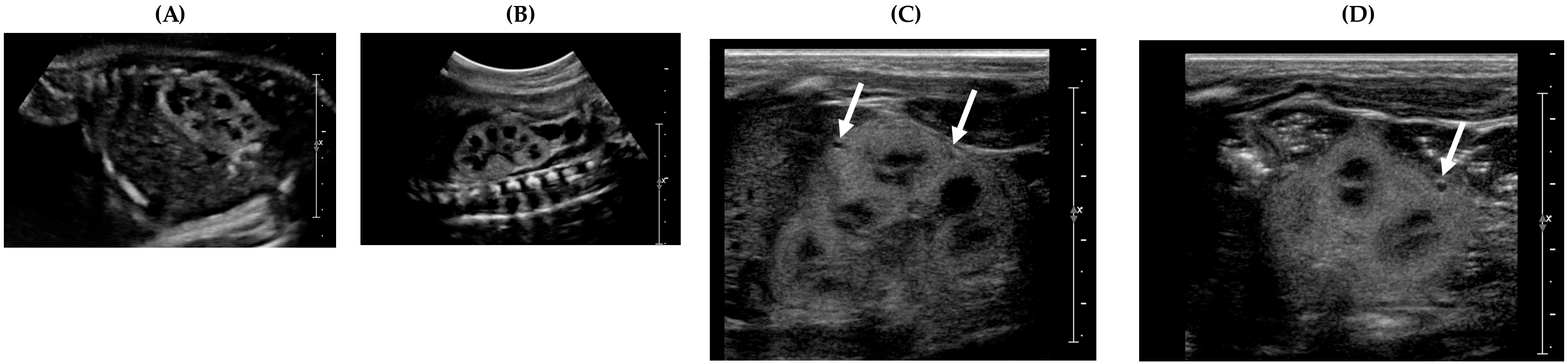

(A, B) Prenatal ultrasound reveals bilaterally hyperechogenic kidneys.

(C, D) Postnatal renal ultrasound demonstrates increased cortical echogenicity of both kidneys with multiple small cortical cysts, suggesting ADPKD. Arrows indicate renal cysts.

Supplementary Figure S5. Case 11 (possible ADPKD)

(A) **(B)** **(C)**

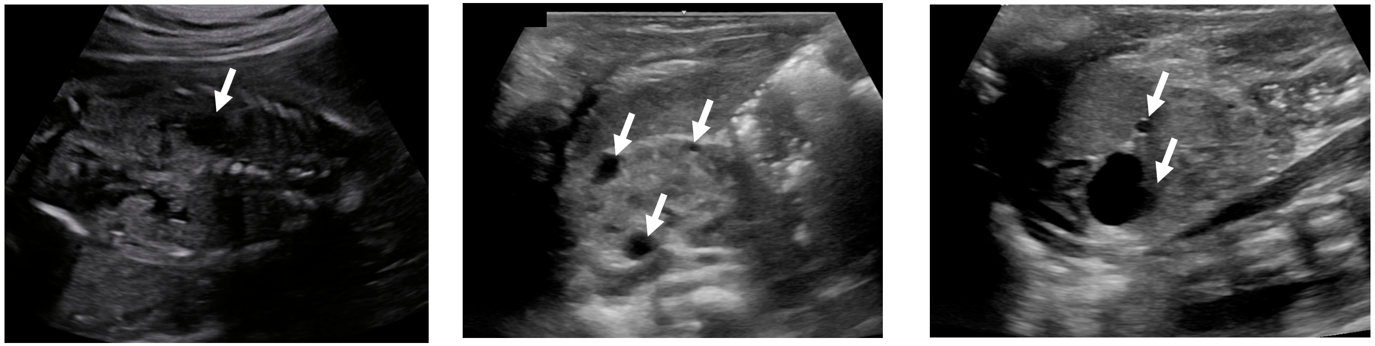

(A) Prenatal ultrasound demonstrates bilaterally hyperechogenic kidneys with a 1 cm anechoic cyst at the upper pole of the right kidney.

(B, C) Postnatal renal ultrasound demonstrates multiple small cortical cysts of variable size in both kidneys, suggesting ADPKD. Arrows point to renal cysts.

Supplementary Figure S6. Case 13 (possible ADPKD)

(A) **(B)** **(C)**

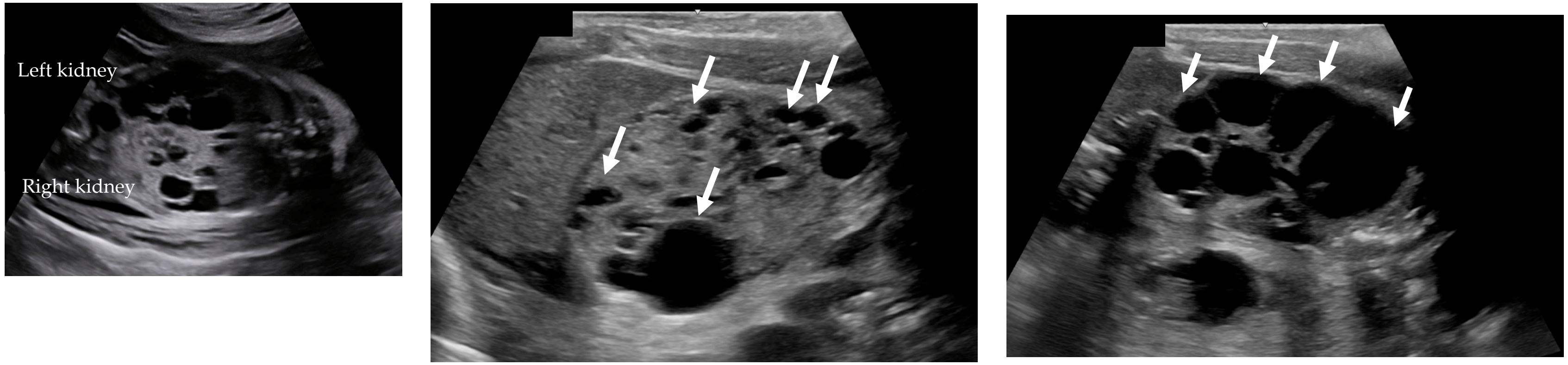

(A) Left kidney, which contains multiple noncommunicating cysts suggestive of multicystic dysplastic kidney (MCDK). Right kidney, which is hyperechogenic with multiple cortical cysts, suggesting ADPKD.

(B, C) Postnatal renal ultrasound performed immediately after birth demonstrates bilateral renal enlargement with increased cortical echogenicity and multiple cortical cysts of variable size. Left kidney (C) shows multiple noncommunicating cysts, a finding more consistent with MCDK. Arrows highlight renal cysts.

Supplementary Figure S7. Case 17 (possible ADPKD)

(A) (B) (C)

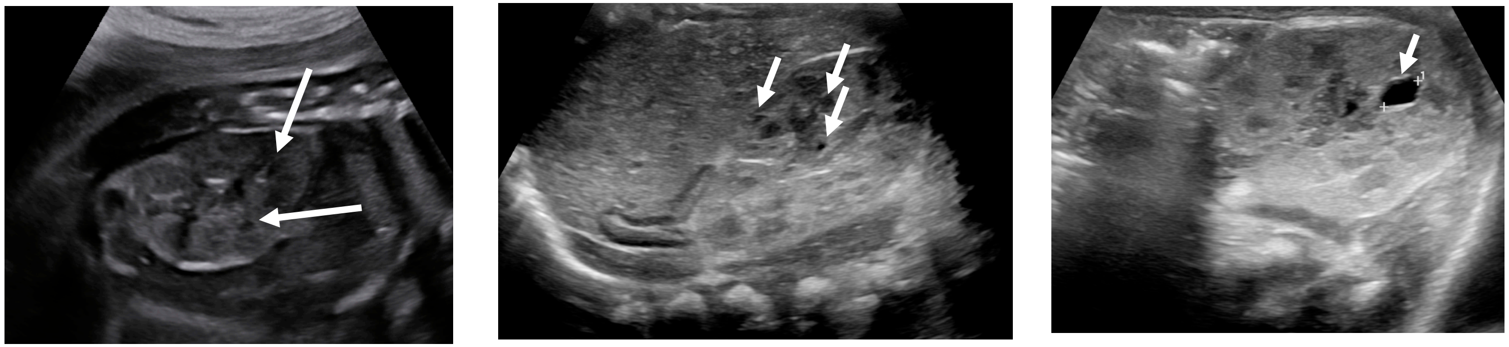

(A) Prenatal ultrasound shows bilateral hyperechogenic kidney with microcystic changes.

(B) Postnatal renal ultrasound indicates a microcystic lesion in the right kidney.

(C) demonstrates a small cystic lesion (0.6 cm) at the mid-pole of the left kidney. Renal cysts are marked by arrows.

Supplementary Figure S8. Case 18 (possible ADPKD)

(A) (B) (C)

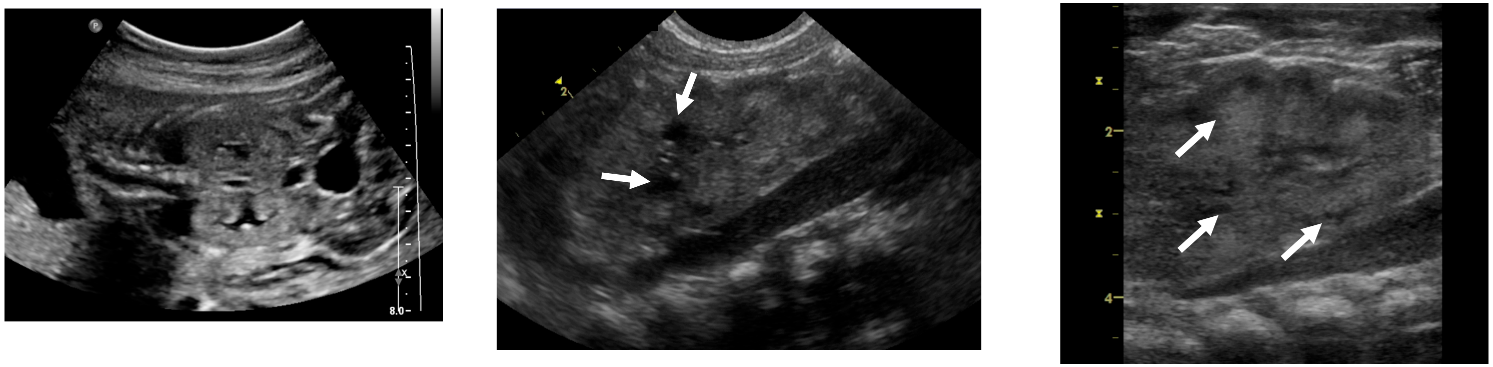

(A) Prenatal ultrasound reveals increased echogenicity of the renal parenchyma with reversed corticomedullary differentiation (CMD).

(B, C) Postnatal renal ultrasound demonstrates bilateral renal cysts and increased medullary echogenicity compared with the cortex, representing a reversed CMD pattern. Arrows indicate renal cysts.

Supplementary Figure S9. Case 19 (possible ADPKD)

(A) **(B)** **(C)**

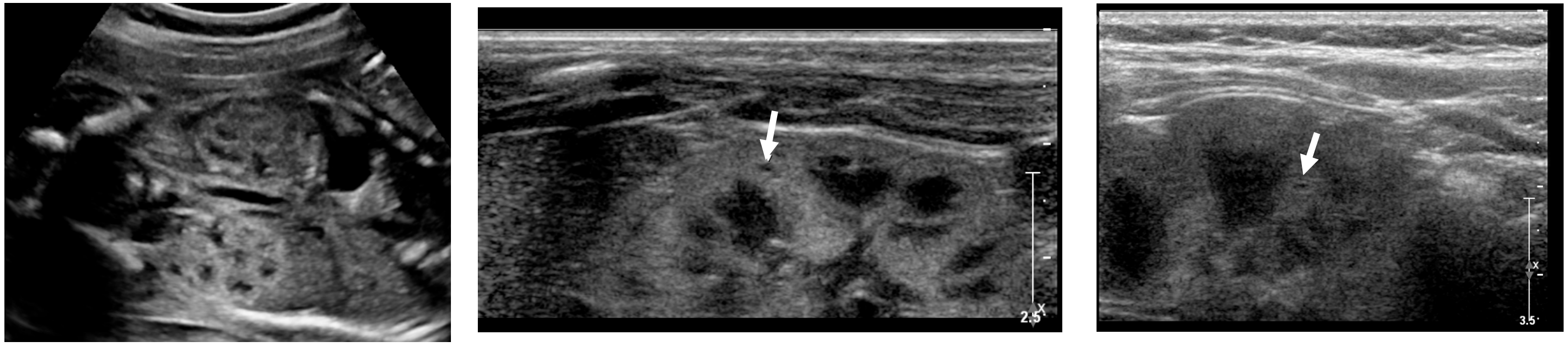

(A) Prenatal ultrasound illustrates bilaterally hyperechogenic kidneys.

(B, C) Postnatal renal ultrasound on the first day after birth demonstrates multiple bilateral cortical cysts and diffusely increased cortical echogenicity in both kidneys. (B) shows the right kidney, and (C) shows the left kidney. Renal cysts are marked by arrows.

Supplementary Figure S10. Case 20 (possible ADPKD)

(A) **(B)** **(C)**

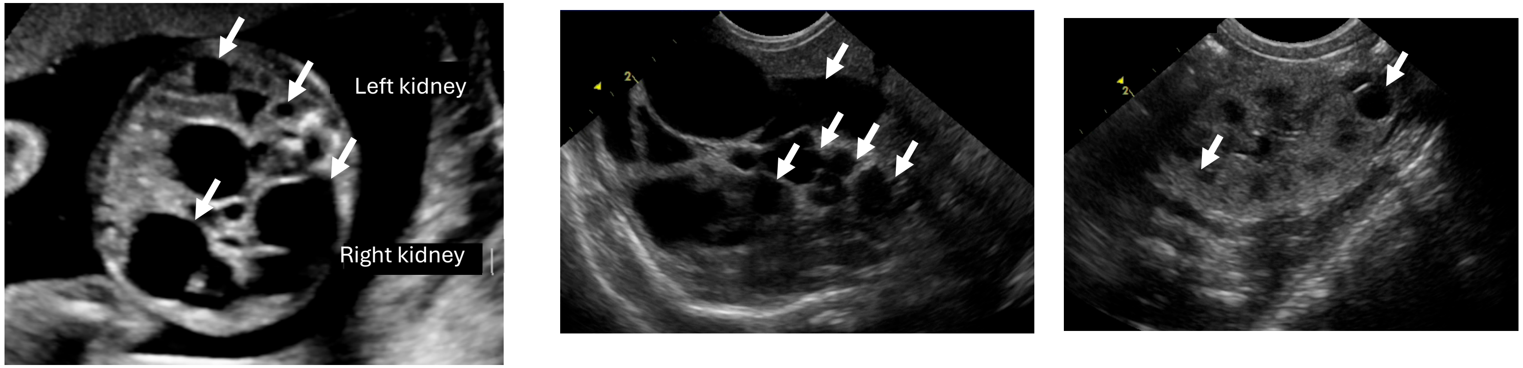

(A) Prenatal ultrasound reveals multiple bilateral renal cysts with hyperechogenic kidneys.

(B, C) Postnatal renal ultrasound demonstrates multiple small cortical cysts of variable size in both kidneys, suggesting ADPKD. Arrows indicate renal cysts.
